# Particle Therapy in Adult Patients with Pelvic Ewing Sarcoma—Tumor and Treatment Characteristics and Early Clinical Outcomes

**DOI:** 10.3390/cancers14246045

**Published:** 2022-12-08

**Authors:** Maximilian P. Schmid, Semi Harrabi, Klaus Herfarth, Øyvind S. Bruland, Thomas Welzel, Thomas Haberer, Malte Ellerbrock, Jürgen Debus, Matthias Uhl, Katharina Seidensaal

**Affiliations:** 1 Heidelberg Ion-Beam Therapy Center, Department of Radiation Oncology, Heidelberg University Hospital, 69120 Heidelberg, Germany; 2 Department of Radiation Oncology, Comprehensive Cancer Center, Medical University of Vienna, 1090 Vienna, Austria; 3 Department of Radiation Oncology, Heidelberg University Hospital, 69120 Heidelberg, Germany; 4 National Center for Tumor Diseases (NCT), 69120 Heidelberg, Germany; 5 Clinical Cooperation Unit Radiation Oncology, German Cancer Research Center (DKFZ), 69120 Heidelberg, Germany; 6 Heidelberg Institute of Radiation Oncology (HIRO), 69120 Heidelberg, Germany; 7 Institute for Clinical Medicine, University of Oslo and Department of Oncology, Oslo University Hospital—Norwegian Radium Hospital, 4950 Oslo, Norway; 8 German Cancer Consortium (DKTK), Partner Site Heidelberg, 69120 Heidelberg, Germany

**Keywords:** proton therapy, carbon ion therapy, radiotherapy, chemotherapy, Ewing sarcoma

## Abstract

**Simple Summary:**

Radiotherapy is a crucial component in the multimodal treatment of Ewing sarcoma; it can be applied for local disease in addition to surgery or as definitive treatment for inoperable disease. Particularly in pelvic Ewing sarcoma, definitive radiotherapy offers the possibility of avoiding extensive and potentially mutilating resections and preserving crucial neurological functions. Adult patients with primary or locally recurrent pelvic Ewing sarcoma treated with curative intent with protons for primary disease and carbon ions for recurrent disease at the Heidelberg Ion-Beam Therapy Center (HIT) were considered for this retrospective analysis. We report excellent dosimetric characteristics in the treatment with protons in comparison to photon IMRT and promising early clinical outcomes. Particle therapy in adult pelvic Ewing sarcoma is shown to be feasible in a consecutive patient cohort. However, further long-term follow-up is needed to assess late toxicity and clinical outcomes.

**Abstract:**

Purpose: To report dosimetric characteristics and early clinical outcomes in patients with pelvic Ewing sarcoma undergoing particle therapy. Methods: Patients ≥ 18 years old with pelvic Ewing sarcoma treated in adjuvant or definitive settings were considered for this retrospective analysis. Proton therapy was carried out with 45–60 Gy (RBE) (1.5–2 Gy (RBE) per fraction) and carbon ion therapy for recurrent disease with 51 Gy (RBE) (3 Gy (RBE) per fraction). Local control (LC), disease control (DC) and overall survival (OS) were calculated using the Kaplan–Meier method. Results: For our sample, 21 patients were available, 18 of whom were treated for primary, 3 for locally recurrent and 16 for inoperable disease. The median CTV and PTV were 1215 cm^3^ and 1630 cm^3^. Median Dmean values for the PTV, bladder and rectum and median V40 Gy for the bowel for patients undergoing proton therapy were 56 Gy (RBE), 0.6 Gy (RBE), 9 Gy (RBE) and 15 cm^3^, respectively. At the end of particle therapy, G 1–2 skin reactions (*n* = 16/21) and fatigue (*n* = 9/21) were the main reported symptoms. After a median follow-up of 21 months, the 2-year LC, DC and OS were 76%, 56% and 86%, respectively. Conclusions: Particle therapy in adult pelvic Ewing sarcoma is feasible and provides excellent dosimetric results. First clinical outcomes are promising; however, further long-term follow-up is needed.

## 1. Introduction

Radiotherapy is a crucial component in the multimodal treatment of Ewing sarcoma that can be applied for local disease as neoadjuvant or adjuvant treatment in addition to surgery or as definitive treatment without surgery. Particularly in pelvic Ewing sarcoma, definitive radiotherapy offers the possibility of avoiding extensive and potentially mutilating resections and preserving important neurological functions. Ewing sarcoma is a rare entity and affects mainly younger patients. Randomized studies of treatment regimens were conducted mainly in children [1,2]. Treating older patients can be challenging since increased chemotherapy-related toxicity can be observed frequently. For that reason, documented data on the details of treatment, outcomes and toxicity in adults are limited. We, therefore, sought to report results in the adult population.

Typical doses of radiotherapy for Ewing sarcomas range from 45 to 60 Gy, which are usually delivered with photons as 3D conformal (3D-CRT) or intensity-modulated radiotherapy (IMRT). In pelvic locations, these target doses can lead to significant exposure of surrounding organs to the dose, such as the urinary bladder, rectum or bowel, with associated treatment-related toxicity. Depending on the configuration and location of the tumor, 3D-CRT generally leads to large high-dose volumes outside of the target volume, whereas IMRT typically enables improved conformality of the high-dose area at the cost of increased volumes exposed to low or intermediate doses of radiation. In contrast to 3D-CRT or IMRT, particle therapy in terms of protons or carbon ions allows, due to its distinct physical characteristics, precise adaptation of the high-dose area while simultaneously reducing low-to-intermediate dose volumes [3]. These dosimetric advantages can reduce treatment-related toxicity, as recently shown for patients undergoing radiochemotherapy for various sites, including pelvic cancers [4]. However, the switch from photon therapy to particle therapy in pelvic Ewing sarcoma implies additional risks, for instance, there may be unexpected toxicity or recurrences due to potential hardly predictable dosimetric variations (e.g., within the spread-out Bragg peak or due to organ movements and air-filled cavities).

Here, we present dosimetric characteristics and first clinical experiences with proton therapy in primary pelvic Ewing sarcoma and carbon ion therapy in locally recurrent pelvic Ewing sarcoma in adult patients.

## 2. Material and Methods

### 2.1. Patients and Treatment Concepts

All eligible patients with pelvic Ewing sarcoma treated between 2013 and 2020 at the Heidelberg Ion-Beam Therapy Center (HIT) were included in this retrospective analysis. Inclusion criteria were age 18 years or older, biopsy-proven primary or locally recurrent disease, no evidence of metastatic disease or limited metastatic disease with good response after chemotherapy and, hence, treatment with (potentially) curative intent.

All patients were staged with MRI for assessment of local disease and CT or PET-CT for assessment of metastatic disease. Overall treatment concepts and chemotherapy regimens were based on the Euro-Ewing 2008 and ISG/ISS 3 and 4 protocols in case of primary disease. Individual chemotherapy regimens, including the combination Irino-/Topotecan and Temozolomide, were followed in cases of locally recurrent disease.

### 2.2. Radiotherapy

Patients with primary disease were treated with proton therapy, whereas patients with locally recurrent disease were treated with carbon ion radiotherapy. The rationale for this choice was that in primary disease, proton therapy was accompanied by concurrent chemotherapy. Protons are considered to be equally effective to photons, in contrast to carbon ions, which have a higher radiobiological effectiveness [4,5]. For these reasons, the dose applied by carbon ions cannot easily be translated into the current treatment protocols.

Patients treated in accordance with the Euro-Ewing Protocol were prescribed total doses of 54–60 Gy (RBE) and 45–54 Gy (RBE), with 1.8–2 Gy (RBE) per fraction daily (five treatments a week) in definitive and adjuvant settings, respectively. Patients treated within the ISG/ISS protocol were prescribed total doses of 54 Gy (RBE), with 1.5 Gy (RBE) per fraction twice daily, in a definitive setting. Patients treated with carbon ion radiotherapy were prescribed 51 Gy (RBE) with 3 Gy (RBE) per fraction. Based on the location of the tumor, patients were positioned in the prone or supine position. For treatment planning, MRI at diagnosis, planning MRI at start of radiotherapy (after completion of induction chemotherapy) and planning CT were fused by rigid image registration based on the bone anatomy. Target volume definition was based on the aforementioned protocols. In short, the gross tumor volume at diagnosis (GTV-init) plus margins was used to create the (extended) clinical target volume (CTV-init) in the first phase of the treatment, followed—if applicable—by a CTV-boost based on the GTV at start of radiotherapy plus margins. The inclusion of whole bones (such as the sacrum) or the whole pelvis in the CTV-init was only considered in cases of skip lesions or disseminated disease within the respective structures. Organ walls (e.g., bladder or bowel) displaced by expansive (non-infiltrating) tumor formations at diagnosis were not included in the CTV. Planning target volumes (PTV) were generated by adding 5 mm margins to the CTVs and 7 mm margins in the beam direction. Bladder, rectum, bowel, femoral heads and cauda (and testicles or uterus, if applicable) were contoured as organs at risk in all patients according to the institutional protocol.

**Figure 1:**
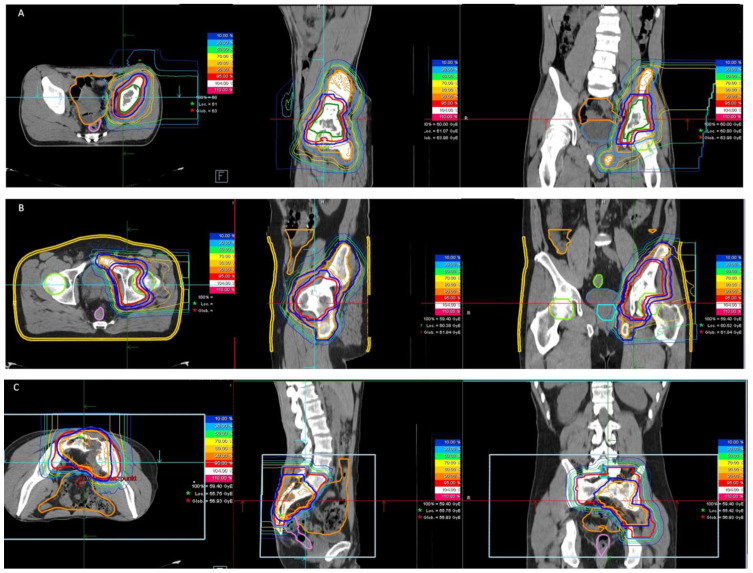
(**A**–**C**): Examples of three patients with primary pelvic Ewing sarcoma undergoing proton therapy.

**Figure 2:**
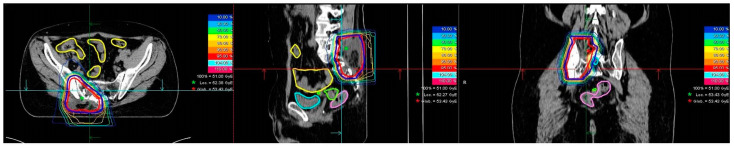
Example of a patient with locally recurrent pelvic Ewing sarcoma undergoing carbon ion radiotherapy.

Treatment planning was performed with syngo RT planning software (Siemens, München, Germany). Proton and carbon ion therapy was performed at HIT with active raster scanning [6,7]. A constant relative biological effectiveness (RBE) of 1.1 was applied for proton beams; for carbon ions, the RBE was calculated with the local effect model (LEM 1). Patient position verification and dose delivery were controlled with daily image guidance using orthogonal X-rays and by forward planning using repetitive CT scans performed during the course of radiotherapy. Examples of proton therapy and carbon ion therapy are shown in Figure 1A–C and Figure 2, respectively.

For comparison, volumetric modulated arc radiotherapy (VMAT) photon plans were created for *n* = 18 patients and optimized aiming at the same clinical goals as the according proton plans. Treatment planning was performed by the planning software RayStation (RaySearch Laboratories) for a linear accelerator (Elekta Versa HD, Stockholm, Sweden).

### 2.3. Data Collection and Study Endpoints

The study was approved by the Ethics Committee of the University of Heidelberg. Data on patient, tumor and treatment characteristics were collected from the medical records and the research platform of the Heidelberg Institute for Radiation Oncology. Morbidity was scored based on CTCAE version 5. Disease outcome was evaluated for local recurrence (LC), distant recurrence and disease control (DC). Local recurrence was defined as any relapse within the pelvis, distant recurrence as any other recurrence and disease control defined by any disease recurrence. Overall survival (OS) was defined by death from any cause. Time-to-event intervals were calculated from the date of biopsy to any of the respective events. Patients without events were censored at the date of last follow-up. The observed differences between the VMAT and proton plans were tested using Student’s two-sided *t*-test. A *p*-value of less than 0.05 was considered to be statistically significant. Descriptive statistics (Excel, Microsoft) and the Kaplan–Meier method (SPSS version 26) were used for analysis.

## 3. Results

### 3.1. Patient Cohort

Overall, 21 patients were available for this analysis. The median age was 23 years. Eighteen patients were treated for primary disease and three patients were treated for locally recurrent disease. Sixteen patients were treated by particle therapy in the definitive setting. The median GTV-init volume was 307 cm^3^. Seventeen patients had a GTV-init > 200 cm^3^. Innominate bones were involved in 14 patients. The median tumor regression after induction chemotherapy was 55%. Two patients presented with distant metastasis at diagnosis. Details are presented in Table 1 and Table 2.

### 3.2. Treatment Characteristics

Treatment settings, corresponding prescribed doses and dose–volume histogram (DVH) parameters are described in Table 3. The median CTV and PTV-init volumes were 1215 cm^3^ and 1630 cm^3^, respectively. The median Dmean for PTV and the rectum, median V40 Gy for the bowel and median Dmax for the femoral head and cauda for patients undergoing proton therapy were 56 Gy (RBE), 9 Gy (RBE), 15 cm^3^, 43 Gy (RBE) and 52 Gy (RBE), respectively. Overall, bladder doses were very low (Dmean: 0.6 Gy) in our cohort in all but two patients (one patient with extraskeletal Ewing sarcoma, V40 Gy: 80%; one patient with large soft tissue component with initial displacement of the bladder, V40 Gy: 42%).

In the VMAT plan comparison for the 18 patients treated for primary disease, DVH parameters were strikingly higher for the organs at riskcompared to the the original proton plans. The Dmean for PTV, bladder and rectum, median V40 Gy for bowel and median Dmax for femoral head and cauda were 53 Gy, 15 Gy, 29 Gy, 59 cm^3^, 45 Gy and 55 Gy, respectively (Table 3).

With regard to the bowel, significant differences were observed for V40 Gy and V30 Gy, which amounted to 15 and 42 cm^3^ for protons in contrast to 59 and 149 cm^3^ for VMAT plans.

### 3.3. Clinical Outcomes

The morbidity spectra at baseline and at the end of radiotherapy are summarized in Table 4. The HIT is a highly specialized center, and many patients travel long distances to receive radiotherapy; consequently, follow-up is often performed closer to home. Although many patients utilized our additional offer to review local MRIs and further imaging examinations, information on toxicity was more difficult to obtain (*n* = 11 of 21 after the end of RT). At baseline, pain (*n* = 9, 43%) and neurological impairment (*n* = 12, 57%) were the main reported symptoms, whereas at end of particle therapy, skin reactions (*n* = 16, 76%) and fatigue (*n* = 9, 43%) were most frequently reported. Slight improvement of neurological functions and pain levels was observed during particle therapy.

After a median follow-up of 21 (3–60) months, two (one in-field, one out-of-field, see below) local recurrences and four distant recurrences were observed. Three patients died of disease. Two patients with recurrence are currently undergoing systemic treatment and one patient with recurrence was lost to follow-up. The corresponding 2-year LC, DC and OS were 76%, 56% and 86%, respectively.

In patients undergoing treatment for primary disease, one local and three distant recurrences were observed (corresponding two-year LC, DC and OS: 83%, 77% and 92%, respectively). The local recurrence occurred in the sacrum in a patient with primary location at the os ilium without evidence of sacral infiltration at diagnosis. By reviewing the target volume and dose distribution in this patient, it was classified as out-of-field recurrence. Locations of distant metastases were bones in two patients and lung and bones in one patient.

All three patients with locally recurrent disease had received previous conventional radiotherapy for primary disease (45, 54 and 59.4 Gy, with 1.8 Gy per fraction). The intervals from the end of primary treatment to the start of second-line treatment were 5, 24 and 36 months. These local recurrences were all considered as in-field recurrences. After re-irradiation with carbon ion radiotherapy, one local and one distant recurrence were observed. A second local recurrence occurred 12 months after end of carbon ion radiotherapy and was classified as field-border recurrence. The patient with distant metastasis (bones) developed disease progression directly after having completed carbon ion radiotherapy.

Comprehensive data on treatment-related morbidity during follow-up (Table 4) were available in 11 patients, comprising skin reactions, fatigue, pain, soft tissue edema, soft-tissue fibrosis, asymptomatic sacral fracture, urinary incontinence and sensory and motoric impairment (last three all evident in a patient treated for locally recurrent disease with consecutive development of local recurrence). Only four patients reported mild gastrointestinal symptoms (3: G1 diarrhea, 1: G1 obstipation) at the end of treatment. Four patients had G1–2 urinary symptoms at the end of treatment, which were dysuria (*n* = 2) and increased frequency (*n* = 2), and one during follow-up (G1 urinary incontinence after re-irradiation in a patient with subsequent development of local recurrence). No grade 3–5 morbidity was observed.

## 4. Discussion

Ewing sarcoma is a rare disease, occurring mainly in children and adolescents, and about 20% of cases are located in the pelvis [1]. Presence of distant metastasis, pelvic location, tumor volumes > 200 cm^3^ or diameter > 8 cm and poor histological response to chemotherapy are all considered as dismal prognostic factors [8,9,10,11,12]. The optimal choice and sequence of local treatment modality in Ewing sarcoma (surgery vs. radiotherapy vs. combination) is still controversially discussed, and the decision on treatment applied is largely influenced by the expected treatment-related sequelae. Surgery is generally favored as local treatment, but resection with adequate margins can be difficult to achieve in the pelvis. The Euro-Ewing 2008 protocol states that inoperability is given if the tumor cannot be assumed to be completely resected or if complete resection results in unacceptable mutilation or is associated with a high risk of serious complications. Within pelvic Ewing sarcomas, sacral tumors appear to have a better outcome than non-sacral tumors [8]. Recent literature indicates that definitive radiotherapy may be the local treatment of choice for sacral tumors, whereas in non-sacral tumors, a combined approach might be beneficial, underlining the overall importance of radiotherapy in pelvic Ewing sarcoma [8,10]. However, pelvic radiotherapy is also associated with significant toxicity, as is well-known for prostate, rectal or gynecological cancers. The limited literature on radiotherapy-related toxicity for pelvic Ewing sarcoma comprises mainly G1–G3 genitourinary, gastrointestinal and musculoskeletal symptoms but also includes severe conditions such as bowel perforation, hemorrhagic cystitis requiring cystectomy and osteoradionecrosis requiring hip replacement [9,13]. Besides other factors such as comorbidity, impact of chemotherapy and bone destruction by the tumor itself, the reason for these toxicity patterns might also be related to the radiation dose distribution. The typically large target volumes pose significant challenges for sparing surrounding pelvic organs with standard 3D-CRT or IMRT. A previously published treatment planning comparison between 3D-CRT and IMRT in eight patients with pelvic Ewing sarcoma revealed acceptable outcomes, but high median doses for the bladder (Dmean 3DCRT: 32 Gy, IMRT: 34 Gy, V40 Gy: 3D-CRT: 41% IMRT: 32%), rectum (Dmean 3D-CRT: 33 Gy, IMRT: 28 Gy; V40 Gy: 3DCRT: 38% IMRT: 9%) and bowel (V40 Gy and V30 Gy: 3D-CRT: 22% and 29% (corresponding to approx. 377 cm^3^ and 497 cm^3^) IMRT: 8% and 16% (corresponding to approx. 137 cm^3^ and 274 cm^3^) [14].

Proton therapy can substantially reduce the dose to organs at risk in comparison to the photon radiotherapy, as shown for various tumor sites [15]. To the best of our knowledge, the current study is the first detailed report of DVH parameters in patients with pelvic Ewing sarcoma undergoing particle therapy. Even though a direct clinical comparison with the aforementioned results from photon radiotherapy is not possible due to different patient cohorts, a clear trend toward improved organ sparing by proton therapy can be derived. In addition, differences in plan robustness should be considered as the high level of precision of particle therapy places special demands on its implementation. The dose distribution of protons is significantly more sensitive to deviations from the planned anatomy in the patient than that of photons. Even small changes in the irradiated tissue, for example, due to positioning inaccuracies, variable fillings of hollow organs such as the bladder or rectum or increased muscle tension of gluteal muscles, can vary the range of the protons or the position of the dose in the patient. Due to the high complexity and sensitivity of particle therapies, quality assurance is significantly more complex than with photons.

The collected DVH parameters from our study can serve as real-world reference values of a consecutive patient cohort treated for pelvic Ewing sarcoma, and may be used to support decision pathways for centers considering referring their patients to a particle therapy facility without the possibility of an individual plan comparison.

In agreement with the dosimetric results, genitourinary and gastrointestinal morbidity were relatively low in our patient cohort. Dose constraints for diarrhea, such as V40 Gy < 124 cm^3^ (prostate cancer) [16]) or V15 Gy < 275 cm^3^ (cervical cancer) [17], are considered relevant. Only one patient (V40 Gy = 199 cm^3^ + V15 Gy = 347 cm^3^) from our cohort violated these constraints. Skin reactions (*n* = 16/21, 76%) and fatigue (*n* = 9/21, 43%) were the predominant symptoms after particle therapy.

In the EMBRACE study, the incidence of ≥G1 fatigue was 69% for patients undergoing radiochemotherapy for cervical cancer [18]. In these patients, large high-dose volumes treated by external beam radiotherapy (e.g., V57 Gy > 182 cm^3^) were independent risk factors for the development of treatment-related fatigue [19]. Accordingly, in our patient cohort, large volumes were treated due to large tumor sizes and the necessary additional margins—the median PTV-init volume was 1630 cm^3^ (treated to a mean prescribed dose of 45 Gy) and the median PTV-boost volume was 655 cm^3^ (treated to a mean cumulative prescribed dose of 59.4 Gy).

So far, the typical total dose applied in cases of definitive radiotherapy in adult Ewing sarcoma is in the range of 45 Gy up to 60 Gy, depending on the anatomical site and size of the tumor. However, a dose–effect relationship has previously been demonstrated [20]. A recent randomized phase three trial (*n* = 95) showed a significantly superior five-year LC rate of 76.5% vs. 49.4% (*p* = 0.02) with dose escalation even up to 70.2 Gy compared to 55.8 Gy, at the expense of a rate of acute radiation dermatitis higher than grade 2 [21].

A structured and comprehensive investigation of the optimal dose according to risk factors will be part of future studies, such as, for example, the iEuroEwing protocol of the Cooperative Ewing Sarcoma Study Group. Our data and plan comparison show that with regard to the perspective of safe dose escalation and the concomitant potential of additional toxicity, a specific focus should be placed on the option of proton therapy, thereby allowing greater normal tissue sparing.

Proton therapy could be performed in all of our patients with primary disease. Yet, the disease outcome and long-term morbidity reported need to be evaluated with caution related to the relatively short follow-up and limited patient numbers. Three-year local tumor control rates in pelvic Ewing sarcoma range from 75 to 100% [9,11,13,22]. The patient and tumor characteristics in our study with two (10%) patients with distant metastasis at diagnosis, three (14%) patients with locally recurrent disease and 17 (81%) patients with GTV-init > 200 cm^3^, in an adult cohort with 55% median tumor regression after induction chemotherapy, point toward a high-risk cohort. Performance of carbon ion radiotherapy for patients with local (in-field) recurrence after previous radiotherapy was feasible and well-tolerated. However, outcomes in these heavily pretreated patients were poor, which indicates the importance of careful patient selection.

The retrospective study design, the small patient cohort and the short follow-up are the main limitations of this study. However, with regard to the rarity of pelvic Ewing sarcoma and the limited literature, in particular, for adult patients, the new perspective on particle therapy provided here appears valuable. 

## 5. Conclusions

Particle therapy in adult pelvic Ewing sarcoma is feasible in a consecutive patient cohort and leads to excellent dosimetric results. The first clinical results are promising; however, further follow-up is needed.

## Figures and Tables

**Table 1:** General patient, tumor and treatment characteristics.

Parameter		(*n*)
Total number of patients (*n*)		21
Median age at diagnosis in years (range)		23 (18–55)
Gender (*n*)		
	male	16
	female	5
Median Karnofsky Index at radiotherapy (range)		90 (60–100)
Local tumor status at diagnosis (*n*)		
	primary	18
	recurrent	3
Location (*n*)		
	innominate bones	8
	sacrum	6
	both (sacrum + innominate)	6
	extraskeletal	1
ESWR1 rearrangement (*n*)		
	yes	12
	no	1
	unknown	8
Distant tumor status at diagnosis (*n*)		
	no metastasis	19
	bone metastasis	1
	bone + lung metastasis	1
Chemotherapy regimen (*n*)		
	Euro-Ewing 2008	13
	ISG/SSG-3	3
	ISG/SSG-4	1
	temodal + irinotecan/topotecan	3
	VAC + IE	1
Radiotherapy setting (*n*)		
	definitive	16
	adjuvant	4
	additive	1
Type of radiotherapy (*n*)		
	proton	18
	carbon ion	3

**Table 2:** Tumor and target volumes.

Parameter	
Median GTV-init in cm^3^ (range)	307 (15–2599)
Median GTV at radiotherapy in cm^3^ (range) *	161 (13–2095)
Median tumor regression after Cht induction in %	55 (15–95)
Median CTV-init in cm^3^ (range)	1215 (207–3385)
Median CTV-boost in cm^3^ (range) *	458 (121–2110)
Median PTV-init in cm^3^ (range)	1630 (337–4798)
Median PTV-boost in cm^3^ (range) *	655 (212–2630)

* Only patients with primary tumor treated in definitive/additive setting.

**Table 3:** Prescribed doses and DVH parameters.

		Proton	VMAT	*p*-Value
Primary tumor treated in definitive setting (protons)		*n* = 15		
	median total prescribed dose (range) in Gy (RBE)	59.4 (54–60)		
	median single prescribed dose (range) in Gy (RBE)	1.8 (1.5 *–2)		
Primary tumor treated in adjuvant setting (protons)		*n* = 3		
	median total prescribed dose (range) in Gy (RBE)	45 (45–54)		
	median single prescribed dose (range) in Gy (RBE)	1.8 (1.8)		
Recurrent tumor treated in definitive/additive/ adjuvant setting (carbon ions)		*n* = 3		
	median total prescribed dose (range) in Gy (RBE)	51		
	median single prescribed dose (range) in Gy (RBE)	3		
		*n* = 18 **		
PTV (median (range) in Gy (RBE)) ***				
	D2%	57 (46–62)	58(46–62)	0.920
	Dmean	56 (44–60)	53 (45–59)	0.130
	D98%	51 (37–58)	45(40–51)	**0.002**
Bladder (median (range) in Gy (RBE))				
	Dmean	0.6 (0–47)	17 (0–54)	**0.034**
	Dmax	31 (0–62)	44 (0–62)	0.288
	D2cc	11 (0–61)	38 (0–61)	0.327
	V40 Gy in %	0 (0–80)	1 (0–100)	0.560
Rectum (median (range) in Gy (RBE))				
	Dmean	9 (0–23)	29 (1–40)	**0.000**
	Dmax	43 (0–60)	53 (2–60)	0.112
	D2cc	32 (0–56)	47 (1–58)	0.063
	V40 Gy in %	0.5 (0–18)	17 (0–59)	**0.005**
Bowel (median (range) in Gy (RBE))				
	Dmax	50 (27–53)	50 (42–57)	0.058
	D1%	41 (11–50)	46(38–52)	**0.014**
	V40 Gy in cm^3^	15 (0–199)	59 (2–198)	**0.015**
	V30 Gy in cm^3^	42 (0–260)	149 (35–394)	**0.001**
	V15 Gy in cm^3^	82 (2–347)	502 (170–1174)	**0.000**
Femoral head in proximity to target volume in Gy (RBE)				
	Dmean	6 (0–58)	24 (0–59)	0.428
	Dmax	43 (0–62)	45 (0–62)	0.640
Cauda in Gy (RBE)				
	Dmax	52 (0–59)	55 (3–62)	0.340
Uterus in Gy (RBE)				
	Dmean	0.2 (0–0.5)	10 (1–23)	**0.022**
	Dmax	8 (0–15)	27 (2–45)	
Testicles in Gy (RBE)				
	Dmax	0 (0–4)	1 (0–23)	**0.049**

* Twice daily; ** patients undergoing proton therapy; *** highest dose level reported, bold values denote statistical significance at the *p* < 0.05 level.

**Table 4:** Morbidity spectra.

		G1	G2	Overall
		*n*	*n*	*n* (%)
Start of radiotherapy (*n* = 21)				
	Skin	0	0	0 (0%)
	Fatigue	1	0	1 (5%)
	Pain	7	2	9 (43%)
	Gastrointestinal	3	0	3 (14%)
	Urinary	1	0	1 (5%)
	Sensory	7	0	7 (33%)
	Motor function	1	4	5 (24%)
End of radiotherapy (*n* = 21)				
	Skin	10	6	16 (76%)
	Fatigue	8	1	9 (43%)
	Pain	5	1	6 (29%)
	Gastrointestinal	4	0	4 (19%)
	Urinary	3	1	4 (19%)
	Sensory	5	0	5 (24%)
	Motor function	2	3	5 (24%)
Follow-up (*n* = 11)				
	Skin	4	0	4 (36%)
	Fatigue	1	0	1 (9%)
	Pain	2	0	2 (18%)
	Gastrointestinal	0	0	0 (0%)
	Urinary	1	0	1 (9%)
	Sensory	0	1	1 (9%)
	Motor function	0	1	1 (9%)
	Soft tissue	3	0	3 (27%)
	Bone	2	0	2 (18%)

## Data Availability

The raw data supporting the conclusions of this article will be made available by the authors, without undue reservation.
